# Long-Term Outcomes of Patients with Primary Brain Tumors after Acute Rehabilitation: A Retrospective Analyses of Factors

**DOI:** 10.3390/life12081208

**Published:** 2022-08-09

**Authors:** Matthew Rong Jie Tay, Justin Desheng Seah, Karen Sui Geok Chua

**Affiliations:** 1 Centre of Rehabilitation Excellence, Tan Tock Seng Hospital Rehabilitation Centre, Singapore 569766, Singapore; 2 Department of Rehabilitation Medicine, Tan Tock Seng Hospital, Singapore 308433, Singapore; 3 Yong Loo Lin School of Medicine, National University of Singapore, Singapore 117597, Singapore; 4 Lee Kong Chian School of Medicine, Nanyang Technological University, Singapore 308232, Singapore

**Keywords:** brain tumors, neuro-oncology, brain cancer, central nervous system tumors, neurological rehabilitation

## Abstract

Although primary brain tumors are relatively rare, they cause significant morbidity and mortality due to the high rates of neurological impairment. The purpose of this study was to examine the physical and functional outcomes of patients with primary brain tumors who had undergone inpatient rehabilitation. This was a retrospective study which recruited 163 patients who had been admitted for inpatient rehabilitation. Rehabilitation outcomes, including the Functional Independence Measure (FIM) and Glasgow Outcome Scale (GOS), were recorded up to 1 year post-discharge. The majority of patients (79.1%) had low-grade (WHO Class I-II) tumors, 35 (21.5%) were diagnosed with GBM and 52 (31.9%) had recurrent brain tumors. Rehabilitation outcomes were sustained, with 125 (76.7%) and 113 (69.3%) patients having a GOS of ≥4 at 6 months and 1 year after discharge, respectively. A GOS of ≥4 at 1 year was negatively associated with high-grade tumors (*p* < 0.001) and radiotherapy (*p* = 0.028), and positively associated with a higher discharge FIM motor score (*p* < 0.001) and the presence of a caregiver after discharge (*p* = 0.034). Our study demonstrates significant positive functional benefits from 4 weeks of inpatient neuro-oncological rehabilitation for patients with primary brain tumors, as well as the importance of supportive care from caregivers.

## 1. Introduction

Although primary brain tumors are relatively rare, they cause significant morbidity and mortality due to the high rates of neurological impairment [1,2,3]. The overall incidence of primary brain tumors is estimated to be up to 25.48 per 100,000 person-years [4], with meningiomas being the most common benign brain tumors (~7.8 per 100,000 person-years) [5] and glioblastomas the most common malignant brain tumors (~3.19 per 100,000 person-years) [6].

In patients with high-grade primary brain tumors, such as glioblastoma multiforme (GBM), the 3-year survival rate has improved marginally from 8.0 to 10.5% in the last decade due to advances in diagnostic and treatment modalities and the use of molecular characterization [7,8,9,10,11]. However, these patients often live with persistent disability. Similarly, patients with low-grade primary brain tumors also have a significant symptom burden and disability compared to the control group of patients with non-CNS cancers and healthy patients [12].

Compared to stroke and traumatic brain injury, the effects of inpatient multidisciplinary rehabilitation on survivorship and functional outcomes in patients with primary brain tumors are not well established. A Cochrane review in 2013, which reported mainly observational studies with low-level evidence, suggested that inpatient or home-based rehabilitation programs may improve functional outcomes [13]. Studies suggest that functional improvements, determined using the Functional Independence Measure (FIM) cognition and mobility scores [14], are consistent across various brain tumor diagnoses, including meningiomas, astrocytomas, GBM and brain metastases [15,16]. Positive factors that have been found to be associated with physical recovery in brain tumor patients include the improvement in motor paralysis after surgery and higher motricity index scores [17,18]. Cognitive recovery in brain tumor patients has also been linked with a younger age and higher education levels [19]. However, a study by Roberts et al. did not find statistical differences in the survival rate of patients who underwent inpatient rehabilitation [20]. 

These impairments may be present simultaneously and include physical and cognitive deficits such as motor weakness or incoordination, cognitive impairments, aphasia, visual field deficits, hearing impairment and seizures. The size, location and grade of the tumor have a major impact on the type and severity of such impairments. They may be secondarily aggravated by sequelae of surgery, radiation, chemotherapy or secondary disease progression [21,22]. As patients can present with a diversity of impairments, an individualized and multidisciplinary rehabilitative approach is required. Rehabilitation of this population is often based on neurorehabilitation principles and the use of specific neuromotor techniques such as neurodevelopmental approaches [23,24]. Patients with brain tumors also have issues such as fatigue, seizures and cognitive dysfunction [25]. Management of fatigue, for example, includes exercise, behavioral and coping strategies and pharmacological treatments to optimize sleep [26]. Most inpatient rehabilitation programs for brain tumors have been described as multidisciplinary, with daily physical therapy, usually over a period of approximately 4 weeks [27,28,29]. 

Most of the current understanding of the rehabilitation of brain tumors is based on Western populations, with a paucity of data on East Asian patients with primary brain tumors. This is despite an incidence of 108,000 cases in a year in East Asia [30]. Reflecting worldwide trends, meningiomas are also the most common tumors in Singapore, comprising 35.1% of brain tumors, while glioblastoma multiforme comprises 9.3% of cancers [31]. Many Western studies have reported the short-term functional outcomes of patients upon discharge from inpatient rehabilitation programs, but not the long-term outcomes [32,33]. Moreover, to date, a study of functional outcomes of brain tumors in Singapore is lacking. 

Hence, this study aims to examine the physical and functional outcomes of patients with primary brain tumors up to 3 years after inpatient rehabilitation and the effects of tumor grade on short- and long-term outcomes.

## 2. Materials and Methods

### 2.1. Study Design

This was a retrospective single-center study of the inpatient and outpatient electronic datasets of all primary brain tumor patients admitted to a single inpatient rehabilitation unit at the Tan Tock Seng Hospital Rehabilitation Centre from January 2013 to December 2020. The study was approved by the National Healthcare Group institutional review board (NHG DSRB 2020/01088), and no patients were recruited prospectively.

### 2.2. Patients

The diagnosis of a primary brain tumor was defined as arising from the brain or meninges, cranial nerves or glands, and confirmed on computed tomography or magnetic resonance imaging of the brain by a neuroradiologist. Patients were classified as having either low-grade (WHO Class I–II) or high-grade (WHO Class III–IV) tumors [34].

Inclusion criteria were datasets of patients who were admitted for inpatient rehabilitation and were transferred directly from acute hospitals following acute treatment of primary brain tumors. Patients who did not complete inpatient rehabilitation, had missing data or had a metastatic brain lesion were excluded. 

Patients were assessed by physiatrists during regular medical rounds in the acute hospital and were transferred for inpatient rehabilitation based on medical stability and suitability for rehabilitation. Patients received standard inpatient neurorehabilitation treatment (3 h/day for 5 days/week), with daily physical therapy, occupational therapy and speech therapy sessions for 1 h each, as well as psychological interventions as needed. Physical therapy included passive/assisted stretching exercises, endurance training, strengthening and balance and gait training. Occupational therapy included cognitive rehabilitation and activities of daily living. Psychological interventions provided counselling and support as required. Muscle tone disorders, e.g., spasticity, were managed via physical therapy techniques (e.g., positioning, stretching, weight bearing, muscle strengthening), pharmacological interventions and injection techniques where appropriate. The patient’s progress and rehabilitation goals were discussed weekly in multidisciplinary rehabilitation team meetings. 

All patients had completed primary surgical resection prior to transfer, with chemotherapy, radiation therapy or immunotherapy performed after completion of inpatient rehabilitation discharge, rather than concurrently, due to logistical limitations. 

### 2.3. Outcome Measures

Admission and discharge functional status were assessed during inpatient rehabilitation by a multidisciplinary team using the Functional Independence Measure (FIM) score [35]. The FIM score is a commonly used 18-item measure of functional status that can be grouped into separate motor (13 items) and cognitive (5 items) domains. It assesses the ability to perform activities of daily living (ADLs) across 6 areas (self-care, sphincter control, transfers, locomotion, communication and social cognition). Each item is scored on a scale ranging from 1 to 7 (from dependent to independent). FIM items are then aggregated into motor and cognitive scores, using the 13 motor items to derive the motor score and the 5 cognitive items to develop the cognitive score (10). A motor FIM score range of 13–91 and a cognitive FIM score range of 5–35 were then obtained. Admission and discharge FIM scores were obtained by trained rehabilitation professionals within 72 h of inpatient rehabilitation admission and discharge from the inpatient rehabilitation unit, respectively. Mean FIM gain (the difference between a patient’s admission and discharge FIM scores) and FIM efficiency (FIM gain divided by rehabilitation length of stay) were also recorded. 

The Glasgow Outcome Scale (GOS) was used to measure disability upon discharge, at 6 months and at 1 year [36]. We classified the patients, based on their GOS scores, into groups with good or poor outcomes. In our study, a poor outcome was defined as a GOS score of 1 (dead), 2 (vegetative state) or 3 (severely disabled), whereas a good outcome was defined as a GOS score of 4 (moderately disabled) or 5 (recovered). We also obtained data on death at 1 year post-discharge, from electronic medical records. Data were retrospectively obtained from outpatient electronic medical records for the 6- and 12-month time points. 

The primary outcome measure was the ability to function independently, defined as FIM >90 upon discharge, which indicates an average of at least FIM level 6 or 7 (modified independence or independent) for each of the FIM categories [37]. A good outcome on the GOS was defined as a GOS score of 4 or 5, measured at 1 year, and whether or not death had occurred at 1 year.

Demographic and clinical data were obtained from medical charts.

### 2.4. Statistical Analysis

We analyzed the data using SPSS version 26.0 (IBM Corp., Armonk, NY, USA). Comparisons of groups in terms of demographic and clinical characteristics were carried out using the t-test, the analysis of variances (ANOVA) or the chi-square test as appropriate. Subset analyses were also carried out for malignant and benign tumors separately.

Logistic regression analyses were used to determine associations with a discharge total FIM score of >90 (indicating mild deficits, without need for physical assistance), with independent variables of age, gender, race, marital status, employment status, Eastern Cooperative Oncology Group (ECOG) performance status [38], lesion side, lesion location, lesion size, tumor grade, tumor recurrence, treatment modalities received (surgical treatment, gross/subtotal resection), acute and rehabilitation length of stay, admission total FIM score and admission motor and cognitive FIM subscale scores. 

Logistic regression analyses were also used to determine a GOS outcome of ≥4 at 1 year, with independent variables of age, gender, race, marital status, employment status, ECOG status, lesion side, lesion location, lesion size, tumor grade, tumor recurrence, treatment modalities received (surgical treatment, gross/subtotal resection, radiotherapy, chemotherapy, Temozolomide), acute and rehabilitation length of stay, caregiver status and FIM scores. 

A significance level of *p* < 0.05 was set for all tests.

## 3. Results

### 3.1. Patient Characteristics

Details of the study population are listed in Table 1. We screened 173 patients, of whom 10 were excluded because they did not complete inpatient rehabilitation. Thus, our study included a total of 163 datasets of patients with primary brain tumors who were admitted for inpatient rehabilitation.

The mean age of these patients was 55.5 (SD = 13.3) years, with a predominance of females (*n* = 102, 62.6%). The population comprised those of Chinese (76.1%), Malay (17.8%) and Indian (6.1%) ethnicity. The majority of patients (79.1%) had low-grade (WHO Class I-II) tumors, 34 (20.9%) were diagnosed with GBM and 52 (31.9%) had recurrent brain tumors. In terms of tumor location, 99 (60.7%) of the brain tumors were supratentorial, 44 (27.0%) were infratentorial and 20 (12.3%) occurred at the base of the skull. All patients underwent surgery, with 125 patients (76.7%) undergoing near-total resections. Additionally, 49 (30.1%) patients received radiotherapy and 17 patients (10.4%) received chemotherapy after their rehabilitation stays.

Of the 35 (21.5%) patients with GBM, 20 patients (12.5%) were diagnosed as IDH wild-type and 15 (9.4%) had unmethylated MGMT status. Among the GBM patients, 14 (8.6%) patients received temozolomide.

A majority of patients (90.8%) were discharged home, and 63 patients (38.7%) did not require a caregiver after completing inpatient rehabilitation (Table 1).

### 3.2. Admission and Discharge Rehabilitation Outcomes

The mean admission FIM total, motor and cognition scores were 71.7 (SD = 23.9), 48.2 (SD = 17.8) and 23.5 (SD = 9.09), respectively. Analysis of the admission scores between recurrent tumors and non-recurrent tumors did not reveal any significant differences in FIM motor scores (*p* = 0.092), FIM cognition scores (*p* = 0.102) or FIM total scores (*p* = 0.065). The mean discharge FIM total, motor and cognition scores were 94.3 (SD = 25.1), 67.0 (SD = 19.1) and 27.4 (SD = 7.65), respectively, for all tumors. There was a mean gain in motor FIM of 18.8 (SD = 12.9) and a mean gain in cognitive FIM of 3.85 (SD = 6.31) from admission to discharge. The mean rehabilitation length of stay was 27.5 days (SD = 22.8) and the mean acute length of stay was 18.6 days (SD = 13.9). 

Further sub-analysis of the rehabilitation outcomes between low-, high-grade and recurrent tumors is shown in Table 2.

A total of 132 patients (81.0%) had a GOS of ≥4 upon discharge. Rehabilitation outcomes were sustained, with 125 (76.7%) and 113 (69.3%) patients having a GOS of ≥4 at 6 months and 1 year after discharge, respectively. Among patients with low-grade tumors, 5 (3.9%) patients demised at 1 year, while among patients with high-grade tumors, 15 (44.1%) patients demised at 1 year (Table 3).

### 3.3. Associations with Rehabilitation Outcomes

For patients who were able to function independently by the time of discharge (discharge FIM >90), multivariate regression analyses revealed a negative association with high-grade tumors (0.252, 0.080–0.797, *p* = 0.019). In contrast, positive associations were found for patients who had premorbid employment (2.67, 1.04–6.90, *p* = 0.042), a higher admission FIM motor score (1.13, 1.07–1.16, *p* < 0.001) and a higher admission FIM cognition score (1.09, 1.03–1.15, *p* = 0.003) (Table 4). 

For patients who had a GOS outcome of ≥4 at 1 year, multivariate regression analyses revealed negative associations with high-grade tumors (0.091, 0.026 = 0.314, *p* < 0.001) and radiotherapy (0.316, 0.113–0.885, *p* = 0.028). In contrast, positive associations were found for patients with a higher discharge FIM motor score (1.094, 1.06–1.13, *p* < 0.001) and the presence of a caregiver upon discharge (3.79, 1.11–12.95, *p* = 0.034) (Table 4). 

For 1 year survival, multivariate regression analyses revealed a negative association with high-grade tumors (0.070, 0.022–0.225, *p* < 0.001) but a positive association with FIM efficiency (2.69, 1.16–6.21, *p* = 0.021).

No significant associations were found with age, chemotherapy, type of surgical resection or use of temozolomide. 

## 4. Discussion

Our study population profile had a predominance of young and economically productive adults typical of the brain tumor survivor population in Asia and worldwide [39,40]. We found that patients with primary brain tumors in our study cohort made significant functional improvements after inpatient rehabilitation. It has been reported that the grade of primary brain tumor does not affect the efficacy of functional improvement during inpatient rehabilitation [41], with Fu et al. reporting comparable FIM efficacies in patients with both low-grade and high-grade astrocytomas [15]. However, compared to those with low-grade tumors, we found that patients with high-grade tumors (WHO grade III-IV) performed significantly worse in both short- and long-term outcomes, attaining significantly lower admission and discharge FIM scores, lower FIM gains and efficiency, a poorer GOS outcome at 1 year and reduced survival at 1 year. Time spent in rehabilitation and acute hospitals was not dissimilar between both groups of tumor grades. 

This difference in our findings could be explained by a significantly greater impairment on admission for both motor and cognitive FIM scores in patients with high-grade tumors compared to low-grade tumors in our study. Conversely, in other studies, both populations had similar impairment levels [15,41]. This could also possibly be explained by the comparatively longer rehabilitation length of stay (nearly 30 days) compared to other studies (22–25 days), although it is acknowledged that funding capitation could be another reason for the shorter length of stay in some countries [13]. Additionally, at present, long-term prognosis for high-grade brain tumors remains unfavorable compared with low-grade tumors, resulting in poorer long-term outcomes [42].

Significant gains post-rehabilitation were reflected in both FIM motor and cognition scores, with an average total FIM gain of 22.6 points. This compares favorably with a total FIM gain of 26.1 and 31.9 points in patients with stroke and TBI, respectively [43]. Smaller gains in FIM motor and cognition scores were reported in patients with high-grade tumors in our study, which were slightly lower than the findings by Roberts et al., who studied a total of 89 patients with newly diagnosed GBM undergoing inpatient rehabilitation. That study reported an improvement in FIM motor and cognition scores of 17.4 and 2.3 points, respectively [20]. Our study on patients with both benign and malignant primary brain tumors found that more significant gains were made in areas of motor function (mean motor FIM improvement of 18.5 points), resulting in improvements in mobility and basic activities of daily living. Smaller, though still significant, gains were observed in cognition (mean cognitive FIM improvement of 3.9 points), which may reflect the limited room for further cognitive improvement in such patients and the predominant emphasis on motor functions in brain tumor rehabilitation programs [16].

Functional improvements also appeared to be sustained, as evidenced by a significant proportion of patients reporting a GOS of ≥4 at 6 months and 1 year. This is consistent with a retrospective study by Yu et al., which found that most patients with both benign and malignant brain tumors had mostly improved or maintained motor and cognitive function at 1–4 years after inpatient rehabilitation [33]. Our findings are also consistent with a recent Cochrane review on brain tumor rehabilitation, which indicated sustained gains at up to 8 months after discharge [13]. Despite a lower survival rate and the possibility of tumor recurrence in patients with primary brain tumors, our findings suggest that long-lasting improvements can nonetheless be achieved. 

Our study found that premorbid employment and higher admission FIM motor and cognition scores were positively associated with a good FIM outcome and independent functioning at discharge from rehabilitation. Premorbid employment may be a reflection of higher premorbid functional status and cognitive reserve. However, brain tumor patients will usually experience cognitive impairment as a result of treatment with surgery, radiation, chemotherapy and/or tumor progression, which can be in the domains of attention, processing speed, memory and executive function [44,45]. This can pose neuropsychiatric barriers to the ability of patients to engage in physical and cognitive rehabilitative programs, and can affect the extent of compensatory or retraining techniques required [46]. Hence, preserving existing cognitive reserve by minimizing the adverse effects of antitumor treatment is important in maximizing the functional benefit of a rehabilitation program [47]. 

Poor determinants of a GOS outcome at 1 year were high-grade tumors and radiotherapy, whereas good determinants were a higher discharge FIM motor score and the presence of a caregiver. Similarly, survival at 1 year was positively associated with high-grade tumors and negatively associated with FIM efficiency. The risk of progression or recurrence in patients with high-grade primary brain tumors is likely to account for a poorer long-term GOS score after discharge, rather than the completeness of resection, which was not a significant variable. The need for radiotherapy may indicate the need to retard the growth of primary brain tumors after incomplete resection, or to treat tumors in unresectable areas, suggesting an association with poorer long-term outcomes [41,48]. Although there are conflicting reports on the effect of concurrent radiation therapy on outcomes [49], our study population did not receive any chemotherapy or radiotherapy during the inpatient rehabilitation phase because our rehabilitation center is located off-site from the acute hospital. 

Most patients with brain tumors are expected to have long-term debilitating sensorimotor, visual–perceptual and cognitive sequelae. With that in mind, it should be highlighted that the presence of a caregiver was a strong predictor of a good GOS outcome at 1 year. The presence of a caregiver may positively affect multiple dimensions of post-discharge function in terms of physical assistance, access to healthcare, response to the patient’s needs and provision of psychosocial support to manage cognitive–behavioral complications. Some studies on cancer patients have hypothesized the positive influence of caregivers on cancer patients’ physical health [50,51], and our findings reinforce the need to provide structured support and guidance for the caregivers (particularly family members) of primary brain tumor survivors after discharge, such as access to caregiver/peer-support networks [52]. 

Several limitations of this study need to be taken into account. The retrospective nature, small numbers, preselection, under-representation of elderly patients and variability in the rehabilitation program over the 8-year study period may limit the generalizability of the findings. Patients who were selected for inpatient rehabilitation may have been of a better functional and medical status, which may account for the observed improvement in rehabilitative outcomes. We also did not utilize the Glasgow Outcome Scale-Extended, which may be more sensitive in determining outcomes in brain injury [53]. Apart from GOS, we also did not collect other long-term quantitative functional measures, such as quality of life measures or FIM scores at 6 months and 1 year, which may shed more light on the physical or cognitive impairments patients encounter after discharge and the relationship between FIM decline and GOS. Although we reported GOS at various time points, we did not have information on the causes of functional deterioration or death, and whether they were cancer-related. Lastly, we did not have detailed information on the specific types of neuromotor or rehabilitative techniques employed in our study population. 

## 5. Conclusions

Our findings lend credence to the significant positive functional benefits of 4 weeks of inpatient rehabilitation for patients with both benign and malignant primary brain tumors. Our findings suggest that the diagnosis of an aggressive primary brain malignancy should not preclude admission to rehabilitation, thus an overly nihilistic approach is discouraged. Despite intensive rehabilitation, only 69.8% and 38.2% of patients with low- and high-grade tumors, respectively, achieved functional independence on discharge, highlighting the challenges in this population. In the majority of our patients, GOS status ≥ 4 was sustained even at 1 year after discharge, while 12.3% demised. With regard to long-term outcomes at 1 year, the presence of a caregiver was a key factor in the ability to sustain good outcomes; hence, due consideration to the training of and long-term support from caregivers, and the prevention of caregiver burnout, are vital elements in the rehabilitation continuum. Further prospective studies are required to understand the interactions between these several factors and long-term quality of life and functional measures.

## Figures and Tables

**Table 1:** Demographic and clinical characteristics of sample (*N* = 163).

Characteristics	
Age, mean (SD)	55.5 (13.3)
Gender (*n*, %)	
- Male	61 (37.4)
- Female	102 (62.6)
Race (%)	
- Chinese	124 (76.1)
- Malay	29 (17.8)
- Indian	10 (6.1)
Marital status (*n*, %)	
- Single	55 (33.7)
- Married	108 (66.3)
Employed (*n*, %)	
- Yes	81 (49.7)
- No	82 (50.3)
Premorbid ECOG (*n*, %)	
- 0	68 (41.7)
- 1	95 (58.3)
Lesion side (*n*, %)	
- Left	61 (37.4)
- Right	69 (42.3)
- Bilateral	33 (20.2)
Lesion location (*n*, %)	
- Frontal/temporal/parietal	99 (60.7)
- Infratentorial	44 (27.0)
- Skull base	20 (12.3)
Lesion size (*n*, %)	
- <3 cm	36 (22.1)
- 3–6 cm	102 (62.6)
- >6 cm	25 (15.3)
Grade (*n*, %)	
- Low grade	129 (79.1)
- High grade	34 (20.9)
Tumor recurrence (*n*, %)	52 (31.9)
Surgery (*n*, %)	
- Surgical biopsy only	6 (3.7)
- Partial resection	32 (19.6)
- Near-total resection	125 (76.7)
Adjuvant Treatment (*n*, %)	
- RT	49 (30.1)
- Chemotherapy	17 (10.4)
- Temozolomide	14 (8.6)
- None	108 (66.3)
GBM subtypes (*n*, %) (*n* = 35)	
- IDH wild-type	20 (12.5)
- Unmethylated	15 (9.4)
Treatment received (*n*, %)	
- Steroid	134 (82.2)
- AED	93 (57.1)
Caregiver on discharge (*n*, %)	
- Resident paid caregiver	25 (15.3)
- Children	12 (7.4)
- Siblings	13 (8.0)
- Spouse	38 (23.3)
- Parents	6 (3.7)
- Institutionalized	6 (3.7)
- None needed	63 (38.7)
Discharge destination (*n*, %)	
- Home	148 (90.8)
- Institution	6 (3.7)
- Other hospitals	9 (5.5)

ECOG: Eastern Cooperative Oncology Group; RT: radiation therapy; GBM: glioblastoma multiforme; IDH: isocitrate dehydrogenase; AED: antiepileptic drugs.

**Table 2:** Admission and discharge rehabilitation outcomes (*N* = 163).

Outcome Variable	All Tumors (*N* = 163)	Sub-Analysis		
		Low-Grade Tumors (*n* = 129)	High-Grade Tumors (*n* = 34)	*p* Value
Admission outcomes				
- FIM motor score, mean (SD)	48.2 (17.8)	49.8 (17.6)	42.0 (17.3)	0.021
- FIM cognition score, mean (SD)	23.5 (9.09)	24.7 (8.67)	18.9 (9.32)	0.001
- FIM total score, mean (SD)	71.7 (23.9)	74.6 (23.2)	60.9 (23.5)	0.003
Discharge outcomes				
- FIM motor score, mean (SD)	67.0 (19.1)	70.3 (17.1)	54.5 (21.3)	<0.001
- FIM cognition score, mean (SD)	27.4 (7.65)	29.0 (6.34)	21.2 (9.05)	<0.001
- Total FIM score, mean (SD)	94.3 (25.1)	99.2 (21.7)	75.7 (28.5)	<0.001
- Total FIM gain, mean (SD)	22.6 (15.9)	24.7 (14.8)	14.9 (17.5)	0.001
- FIM efficiency, mean (SD)	1.08 (0.898)	1.20 (0.858)	0.647 (0.924)	0.001
- Total FIM score >90, *n* (%)	103 (63.2)	90 (69.8)	13 (38.2)	0.001
Length of stay				
- Acute hospital, mean (SD)	18.6 (13.9)	18.9 (17.8)	20.2 (15.2)	0.705
- Rehabilitation, mean (SD)	27.5 (22.8)	28.4 (24.7)	23.3 (11.6)	0.242
Long-term outcomes				
- GOS ≥ 4 at 1 year, *n* (%)	113 (69.3)	105 (81.4)	8 (23.5)	<0.001
- Survival at 1 year, *n* (%)	143 (87.7)	124 (96.1)	19 (55.9)	<0.001

FIM: Functional Independence Measure; GOS: Glasgow Outcome Scale.

**Table 3:** GOS outcomes by time point (*N* = 163).

GOS Discharge	Discharge	6 Months	1 Year
Low grade (*n* = 129)			
- 1	0	2 (1.6)	5 (3.9)
- 2	2 (1.6)	1 (0.8)	1 (0.8)
- 3	15 (11.6)	15 (11.6)	18 (14.0)
- 4	51 (39.5)	47 (36.4)	42 (32.6)
- 5	61 (47.3)	64 (49.6)	63 (48.8)
High grade (*n* = 34)			
- 1	0	2 (5.9)	15 (44.1)
- 2	2 (5.9)	4 (11.8)	1 (2.9)
- 3	12 (35.3)	14 (41.2)	10 (29.4)
- 4	12 (35.3)	10 (29.4)	5 (14.7)
- 5	8 (23.5)	4 (11.8)	3 (8.8)

GOS: Glasgow Outcome Scale.

**Table 4:** Regression analyses of significant factors associated with short- and long-term outcomes.

Variables	OR (95% CI)	*p* Value
Discharge Total FIM score >90		
High-grade tumors	0.252 (0.080–0.797)	0.019
Premorbid employment	2.67 (1.04–6.90)	0.042
Admission FIM motor score	1.13 (1.07–1.16)	<0.001
Admission FIM cognition score	1.09 (1.03–1.15)	0.003
GOS outcome of ≥4 at 1 year		
High-grade tumors	0.091 (0.026–0.314)	<0.001
Radiotherapy	0.316 (0.113–0.885)	0.028
Discharge FIM motor score	1.094 (1.06–1.13)	<0.001
Caregiver	3.79 (1.11–12.95)	0.034
Survival at 1 year		
High-grade tumors	0.070 (0.022–0.225)	<0.001
FIM efficiency	2.69 (1.16–6.21)	0.021

FIM: Functional Independence Measure; GOS: Glasgow Outcome Scale.

## Data Availability

The data presented in this study are available on request from the corresponding author.
